# Abstract of Minutes of the Atlanta Medical Society

**Published:** 1867-10

**Authors:** 


### ARTICLE II.

### *Abstract of Minutes of the Atlanta Medical Society.*

ATLANTA, FEB. 5. 1867.

On the call for reports of cases,

*Dr. Cameron* reported a case of chronic enlargement of the tonsils and uvula, in a man thirty years old. Nitrate of Silver had been used for several weeks without benefit. A few applications of Creasote gave prompt and decided relief.

*Dr. O'Keefe* regarded this an interesting case. He had frequently met with such difficulties in children, and had found them difficult of cure, before the age of puberty, when the enlargement generally disappears, spontaneously. He had seen this trouble associated with suppressed menstruation. His treatment has generally been excision.

*Dr. Johnson* had not been uniformly successful in his treatment of this affection. His experience would go to establish the fact that girls, from six to twelve years old are more subject to it, as the result of vicissitudes in the weather, or sudden exposure to dampness, or a cool current of air, when heated. His attention had not been called to it as the result of uterine trouble, but believes it sometimes due to constipation. In the treatment he has generally relied on cauterants, blisters, &c., but from the report of Dr. Cameron, is disposed to think favorably of Creasote.

*Dr. W. F. Westmoreland* alluded to the effects of Creasote in diphtheria as an evidence of its peculiarly alterative or cauterant action, from which it is not unreasonable to expect benefit in cases of chronic enlargement, such as reported by Dr. Cameron.

*Dr. O'Keefe* reported the following history of a case: Was consulted, six weeks since, in regard to the condition of a lady, who says she has suffered for three months previously to her last four confinements, with what seemed to be a separation of the pelvic bones at the sacro-iliac symphesis. She reports that the grating was distinct, and perceptible to by-standers—that she is usually confined to bed two months after delivery, on this account—the movement of the bones in attempting to walk producing a faintness which compels her to lie down. One week since, this lady was confined, and had a short and easy labor. During the labor an examination, with the view of ascertaining the condition of the pelvis, was made, without a satisfactory result. She now complains of the grating sensation, as usual after delivery. He desires the opinion and experience of members on this subject.

*Dr. Johnson* had recently met a lady, who informed him that fifteen months since she was confined, and, from supposed mobility of the pelvic bones, was unable to walk for six weeks afterward.

*Dr W. F. Westmoreland* thought that a properly applied bandage, so as to keep the articulating surfaces in apposition, would very much facilitate the restoration of these bones to their natural union and strength.

*Dr. Watkins*, by invitation present at the meeting of the society, mentioned a case of this luxation, in which the bandage proved useful.

*Dr. Douglas* alluded to a case of sacro-iliac luxation occurring in labor, followed by caries of the bones.

*Dr. Word* reported a case of supposed gastrodynia. For fifteen years the patient has suffered pain in the epigastric region, at intervals of two or three months. Recently these attacks occur daily, with rigors, followed by profuse perspiration. A gnawing sensation and pain are felt in the stomach, with occasional flatulence and vomiting after eating. The nausea and rejection of food is probably attributable to the secondary effects of opiates, which are required to alleviate the suffering. Pepper and alcoholic drinks effectual in relieving the pain for a short time. Blisters do not give permanent relief. He solicited of members their opinion of the disease, and its proper treament.

*Dr. Stacy* thought the symtoms detailed by Dr. Word may be the result of biliary calculi passing along the ducts.

*Dr. Word* had not discovered any jaundiced appearance, or any other symtom, which, in his opinion, would warrant the supposition of Dr. Stacy.

Dr. Johnson had seen a somewhat similar case in the person of a lady fifty years old; for which various remedies had been used for some time with no benefit. Finally, it being ascertained that constipation was a constant symptom, a cathartic of Calomel, Croton Oil and Colocynth was used with temporary relief. Iron, Quinine, Bismuth, and Hyoscyamus, were used with seeming advantage and Belladonna with decided benefit. The treatment was successfully concluded by a visit to chalybeate springs.

Dr. Douglas thought the case reported by Dr. Word, in all probability, depended on the passage of calculi. This he inferred from the repeated attacks. Some inflammation, probably as the result of the calculi, may have existed.

Dr. O'Keefe gave the following history of a case having somewhat similar symptoms: A lady suffered with severe pain in the stomach, at intervals of one, two or three months. These paroxysms became more frequent, until, in the latter part of her life, the suffering was almost constant. Relief was most readily obtained from the use of Chloroform. Post-mortem examination revealed a cancerous condition of the pancreas. The stomach and duodenum were healthy. Six of the family had previously died of Phthisis.

---

